# Ocular Manifestations of Alport Syndrome: Report and Comparison of Two Cases

**DOI:** 10.7759/cureus.47373

**Published:** 2023-10-20

**Authors:** Meisam Sargazi, Shima Dehghani, Mina Dahmardeh, Seyed Omid Mohammadi

**Affiliations:** 1 Ophthalmology, Zahedan University of Medical Sciences, Zahedan, IRN; 2 Ophthalmology, Burnett School of Medicine at Texas Christian University, Fort Worth, USA

**Keywords:** sensorineural deafness, progressive myopia, lenticonus, retinal thinning, alport syndrome

## Abstract

We report two cases of Alport syndrome and compare the clinical presentations and imaging findings in these cases. The clinical examination consisted of best-corrected visual acuity (BCVA), direct ophthalmoscopy, and slit-lamp examination. Macular optical coherence tomography (OCT) and anterior segment OCT (AS-OCT) and were utilized to document the details of the anterior and posterior segment pathologies. In order to evaluate systemic presentations of Alport syndrome, nephrology, and otolaryngology were consulted for each patient. In this study, the first case was a 27-year-old female with progressive myopia, anterior lenticonus, and temporal retinal thinning found in the ocular examination that led to the diagnosis of Alport syndrome. She underwent clear lens extraction and intraocular lens implantation, restoring acceptable visual acuity. The second case was a 20-year-old male patient with low visual acuity, severe bilateral anterior lenticonus, bilateral cataract, and temporal retinal thinning. The patient later developed renal failure and is a candidate for kidney transplantation. In this case report, progressive renal failure, hearing loss, and ocular abnormalities were all observed. This is consistent with previously reported cases given the typical characteristics of Alport syndrome, a rare inherited disease. The severity of those characteristics was higher in the male subject, a finding also consistent with prior reports indicating that males are usually affected more frequently and more severely than females, given that Alport syndrome is generally inherited as an X-linked disorder. Additionally, anterior segment and macular OCTs seemed to be of considerable significance in the early diagnosis of Alport syndrome given the typical ocular manifestations e.g. anterior lenticonus or temporal retinal atrophy.

## Introduction

Alport syndrome is a rare genetic disease with a prevalence of one case per 5,000-53,000 live births. The main abnormality in this syndrome is the deficient synthesis of type IV collagen, which is the principal component of basement membranes throughout the human body [1-3]. The disease is characterized by progressive nephritis, sensorineural hearing loss, and ocular abnormalities [1]. In most (65%) of the patients, the condition is inherited as an X-linked disorder therefore the frequency and severity of symptoms tend to have significant differences between male and female patients. Homozygote males are usually severely affected, as approximately all affected males eventually develop renal failure by the age of 20 years and have high tone sensorineural deafness [3,4].

The characteristic ocular features of Alport syndrome are corneal opacities, anterior lenticonus, cataract, central peri-macular, peripheral coalescing fleck retinopathies, and temporal retinal thinning. Rarely, patients may also have posterior polymorphous corneal dystrophy and giant macular holes that impair vision [4]. Upon seeing the ocular findings above in a patient, the physician should be highly suspicious of the diagnosis of Alport syndrome [5].

Among the ocular findings, the anterior lenticonus is the pathognomonic feature of Alport syndrome. This condition occurs in up to 20% of X-linked Alport syndrome patients. It is not present at birth but appears later and worsens with age. It manifests as slowly progressive vision deterioration due to progressive myopia. In cases with a severe decline in vision quality, surgical treatment may be needed to restore vision [4,6].

Herein, we report two cases of Alport syndrome, a male and a female. The study discusses and compares the differences in ocular manifestations of Alport syndrome between male and female patients and also reports successful vision restoration using clear lens phacoemulsification and intraocular lens implantation in one of the patients. Additionally, we present the anterior segment optical coherence tomography (AS-OCT; Tomey Casia 2, Nagoya, Japan) images of the anterior lenticonus in both patients, showing a particular type of severity in the male patient.

## Case presentation

Case 1

A male patient in his 20s was referred to our clinic by an internist. He had been hospitalized in the internal medicine department due to renal dysfunction in the setting of previously diagnosed Alport syndrome. He had no history of kidney disease in the family. The patient himself had been hospitalized several times due to renal dysfunction. His surgical history was negative. A systemic examination confirmed he was completely deaf in both ears.

In ocular examination eye movements were normal. No nystagmus was observed. Best-corrected visual acuity (BCVA) was 20/100 in the right eye and 20/70 in the left eye. The patient’s manifest refraction was -19.50 sphere in the right eye and -18.50 sphere in the left eye.

On slit-lamp examination, the cornea and anterior chamber were normal in both eyes. A bilateral oil droplet reflex was seen and optical section illumination through the lens revealed severe anterior lenticonus in both eyes. Intraocular pressure was 14mmHg bilaterally. Posterior segment examination revealed numerous dots and flecks in the peri-macular region (fleck retinopathy).

**Figure 1:**
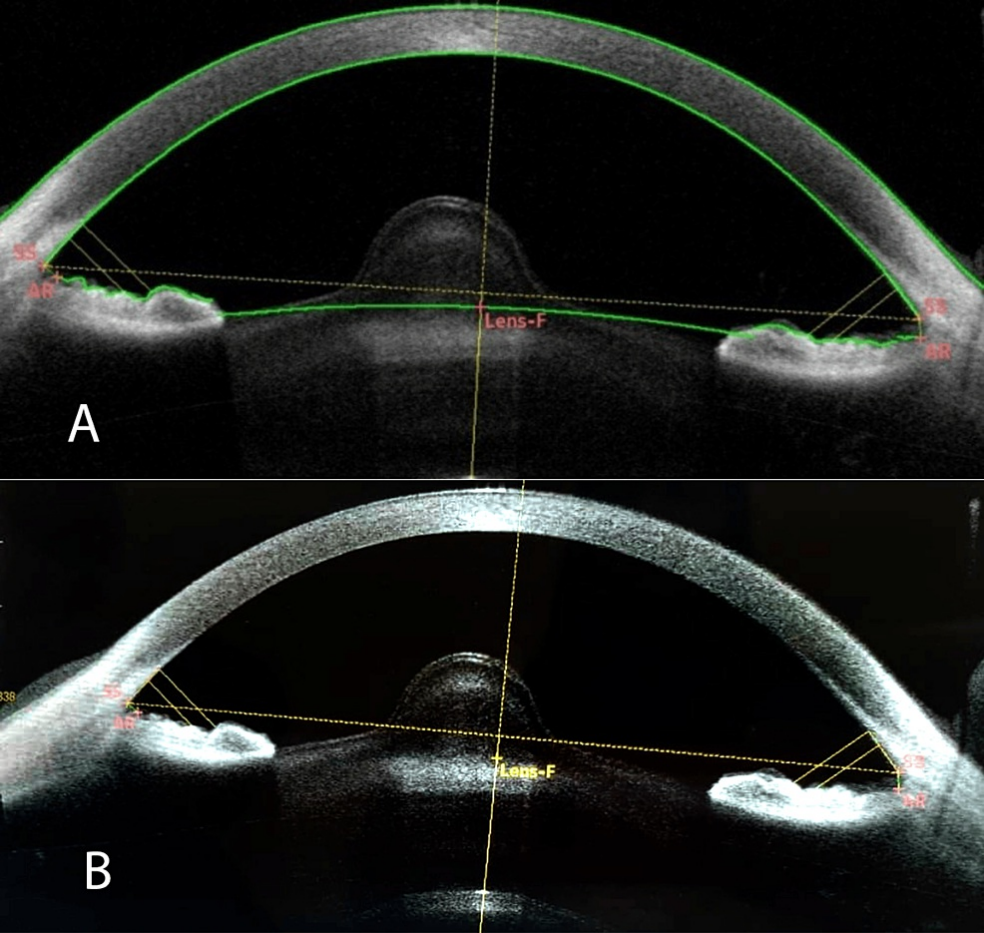
Anterior segment optical coherence tomography: A & B right and left eye, respectively, showing very severe anterior lenticonus in the male patient.

**Figure 2:**
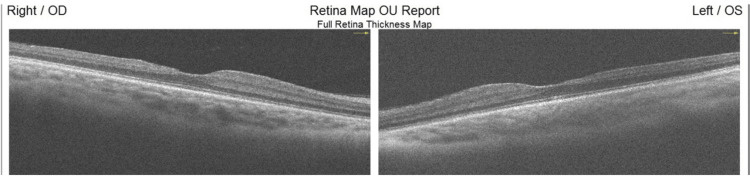
Macular optical coherence tomography: Bilateral temporal retinal thinning in the male patient

AS-OCTs show bilateral severe anterior lenticonus in the male patient (Figure 1). Macular OCT revealed temporal retinal thinning and atrophy in both eyes of the male patient (Figure 2).

Case 2

A female in her 20s came to our clinic for refractive surgery. The patient complained of progressive myopia that had made her change her glasses frequently. She had no past medical history of systemic diseases or consumption of drugs. Her familial history of renal/ocular diseases or hearing loss was negative. A systemic examination revealed a sensorineural hearing loss in her left ear.

On external ocular examination, the patient’s eye movements were normal. BCVA was 20/70 in the right eye and 20/40 in the left eye. The patient's manifest refraction was -7.50/-1.00 ×160 in the right eye and -6.00/-1.25×175 in the left eye.

**Figure 3:**
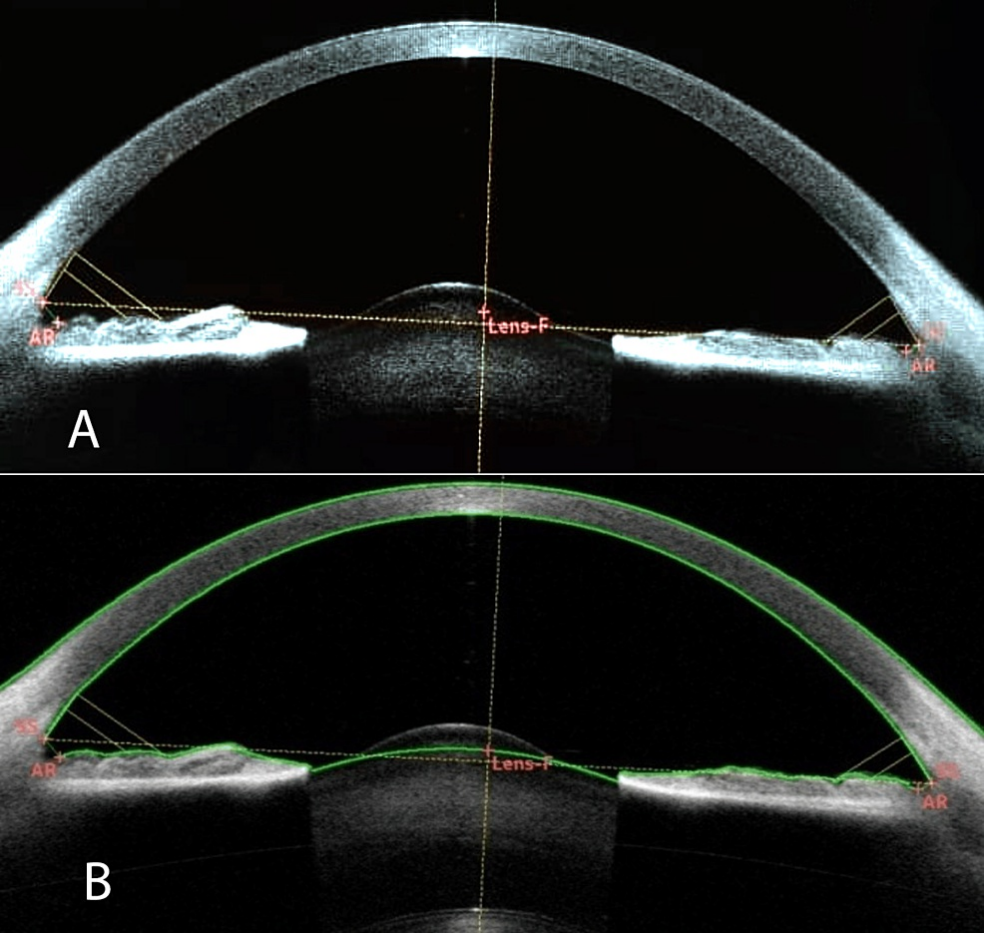
Anterior segment optical coherence tomography: A & B right and left eye, respectively, showing mild anterior lenticonus in the female patient

Slit-lamp examination of the eyes was within normal limits except for the anterior and posterior lenticonus in both eyes. Intraocular pressure was 17mmHg bilaterally. Direct ophthalmoscopy revealed bilateral oil droplet reflex. Funduscopic findings of both eyes were normal. AS-OCT of both eyes of the female patient presented mild anterior lenticonus (Figure 3).

**Figure 4:**
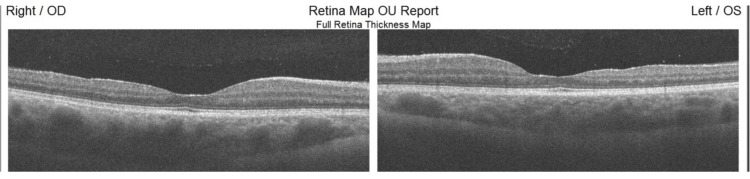
Bilateral temporal retinal thinning in the female patient

In macular OCT, temporal retinal thinning and atrophy were observed in both eyes of the female patient (Figure 4). Based on the clinical findings a possible diagnosis of Alport syndrome was considered. To confirm that, the patient was referred to a nephrologist. In her urine analysis, mild proteinuria was revealed. A kidney biopsy was then performed and confirmed the diagnosis of Alport syndrome. The patient was prescribed angiotensin receptor blocker medication for proteinuria.

As the patient’s lenticonus had caused significant vision loss we decided to proceed with clear lens extraction and intraocular lens implantation. Two weeks after surgery uncorrected distance visual acuity was 20/20 in both her eyes.

## Discussion

Alport syndrome is an inherited disorder of collagen that mainly affects the kidney, eye, and cochlea. The disease is genetically heterogeneous and has variability in its clinical and pathological manifestations. Approximately 65% of the cases of Alport syndrome are inherited as an X-linked disorder. In about 15%, the transmission is autosomal recessive and about 20% of transmission is autosomal dominant. Additionally, rarely patients tend to have no family history. Generally, Alport syndrome affects boys more than girls [3].

The organ affected most significantly by Alport syndrome is the renal system. Hematuria is the earliest clinical sign. This may be the primary presentation of the syndrome in children. As these patients get older, they begin to show additional signs of kidney disease, such as proteinuria and high blood pressure. Both cases presented here had kidney involvement. Boys with X-linked Alport syndrome may develop renal failure by their teenage years or early adulthood [7]. The first case (male patient) received a kidney transplant one month after referral to our clinic due to renal failure, which may be indicative that our case might have had the X-linked type of the disease. Generally, most girls with X-linked Alport syndrome do not develop renal failure and tend to have a milder renal involvement, usually as microscopic hematuria [4,6]. However, they are at an increased risk of developing renal function impairment compared to the normal population [7]. In our second case (female patient), renal involvement was just as mild as proteinuria.

The auditory presentation of bilateral, symmetrical hearing loss in Alport syndrome is quite significant. Most of the patients experience hearing loss by the age of 10. The severity of the auditory manifestations appears to be parallel to the severity of the renal involvement. Females with Alport syndrome are again usually less severely affected and the deficit is usually non-progressive [8]. In our report, the male patient had a more severe sensorineural hearing loss, based on otolaryngology consultation, compared to the female patient. Some studies have found that hearing loss in Alport syndrome is mainly caused by abnormalities in cochlear structures, as the latter has a similar structure (type 4 collagen) to the glomerular basement membrane [8].

Ocular anomalies have been reported in 9% to 82% of Alport syndrome patients [9,10]. They are rare in childhood and they increase in frequency and severity with age. The types of ocular defects described mostly involve the lens and retina, less likely to affect the cornea [11].

Anterior lenticonus is an abnormality in the shape of the lens and one of the most common ocular abnormalities in these patients. It occurs in about 15% to 20% of patients with X-linked and autosomal recessive Alport syndrome. People with anterior lenticonus may show a slowly progressive deterioration of vision (progressive myopia) requiring patients to change the prescription of their glasses frequently. This condition may also lead to cataract formation [12,13]. The male patient reported here had a more severe anterior lenticonus compared to the mild anterior lenticonus observed in the female patient. In this study, we presented the anterior segment OCT image of a particular bilateral severe anterior lenticonus in our male patient. To the extent of our literature review, this type and severity of anterior lenticonus have not been reported. There are not many reports providing anterior segment OCT images and the exact morphological changes in the lens structure (Figures 1, 3) [4,14].

Can et al. reported successful cataract surgery in a 22-year-old Alport patient in 2008 [15]. Similarly, our female patient also gained full visual recovery after clear lens extraction and intraocular lens implantation.

Most patients with Alport syndrome have abnormal pigments in the retina called fleck retinopathy. This condition does not affect visual acuity [16]. In this report, the male patient had evidence of fleck retinopathy and the female fundus was normal. Both of our cases had temporal retinal atrophy that was shown in macular OCT. Temporal retinal atrophy in Alport syndrome has also been reported in two other studies as a new finding in Alport syndrome patients [14,15].

Recurrent corneal erosion, posterior polymorphous corneal dystrophy, and cataracts are other ocular abnormalities that can occur in patients with Alport syndrome [14,17]. None of our patients had signs of corneal dystrophy, but bilateral cataracts were observed in the male patient.

It is important to diagnose Alport syndrome in the early stages of the disease, particularly in female patients. Because of the less severe presentation of the disease, Alport syndrome might remain unrecognized until the disease has already advanced to the more severe stages of renal and ocular involvement. This condition needs to be effectively managed by a combined approach from different specialties including nephrology, otolaryngology, and ophthalmology. Early ophthalmic examination and early ophthalmological interventions such as clear lens extraction or cataract surgery could be carried out to improve vision and avoid amblyopia, all eventually leading to a better visual prognosis for these patients.

## Conclusions

Alport syndrome is a rare genetic disease characterized by deficient synthesis of type IV collagen, leading to a range of clinical manifestations affecting the kidneys, hearing, and vision that mostly affect males more severely. Ocular abnormalities in Alport syndrome include anterior lenticonus, fleck retinopathy, and temporal retinal thinning. Patients may benefit from successful vision restoration through clear lens extraction and intraocular lens implantation, highlighting the potential benefits of early ophthalmological intervention in improving visual outcomes for individuals with Alport syndrome.
